# Supplementary material for: A Weibull mixture cure frailty model for high-dimensional covariates
Source: Stat Methods Med Res. 2025 Mar 31;34(6):1192–218. doi: 10.1177/09622802251327687 (PMC12209551; doi:10.1177/09622802251327687)
Supplement: sj-pdf-1-smm-10.1177_09622802251327687 - Supplemental material for A Weibull mixture cure frailty model for high-dimensional covariates [file sj-pdf-1-smm-10.1177_09622802251327687.pdf]

Supplementary Materials to the paper
A Weibull Mixture Cure Frailty Model for High-dimensional Covariates

Fatih Kızılaslan, David Michael Swanson, Valeria Vitelli

1 Additional tables for the Simulation Study

In Tables S1 and S2, we present the prediction performance for the regression coefficients and the uncured rate estimate performance for the simulated scenarios with correlation $\rho = 0.2$ and 0.5.

Table S1: Simulation studies results for $\mathbf{b}_p$, $\boldsymbol{\beta}_p$ and $\pi(\mathbf{z})$ when $\rho = 0.2$

		$\boldsymbol{\beta}_p$		$\mathbf{b}_p$		$\pi(\mathbf{z})$	
v	Method	RME(SD)	ERR (SD)	RME(SD)	ERR (SD)	Bias	MSE
0.5	A1	1.225 (0.254)	1.227 (0.258)	0.320 (0.522)	0.321 (0.522)	**0.009**	0.067
	A2	1.340 (0.642)	1.342 (0.647)	0.330 (0.532)	0.330 (0.532)	0.014	0.071
	A3	1.415 (0.965)	1.417 (0.968)	0.331 (0.534)	0.332 (0.534)	0.013	0.072
	A4	1.432 (0.984)	1.434 (0.986)	0.332 (0.535)	0.332 (0.535)	0.013	0.072
	B	1.191 (0.050)	1.191 (0.050)	0.335 (0.535)	0.335 (0.535)	0.238	0.130
	C	1.428 (0.323)	1.428 (0.321)	**0.303** (0.506)	**0.304** (0.506)	-0.020	**0.057**
	D	**1.125** (0.125)	**1.127** (0.123)				
1	A1	0.817 (0.127)	0.818 (0.126)	**1.385** (2.632)	**1.387** (2.633)	**0.007**	0.085
	A2	0.803 (0.147)	0.804 (0.147)	1.513 (2.881)	1.514 (2.881)	0.022	0.099
	A3	**0.785** (0.157)	**0.786** (0.156)	1.517 (2.922)	1.518 (2.922)	0.024	0.100
	A4	0.789 (0.161)	0.790 (0.160)	1.512 (2.909)	1.513 (2.909)	0.023	0.101
	B	1.059 (0.028)	1.059 (0.027)	1.816 (3.405)	1.816 (3.405)	0.129	0.156
	C	1.082 (0.051)	1.084 (0.051)	1.411 (2.919)	1.413 (2.920)	-0.036	**0.082**
	D	0.851 (0.087)	0.852 (0.086)				
1.5	A1	0.727 (0.089)	0.728 (0.089)	2.040 (4.292)	2.041 (4.292)	**0.009**	**0.078**
	A2	0.654 (0.129)	0.654 (0.129)	**2.033** (4.343)	**2.034** (4.343)	0.026	0.089
	A3	**0.634** (0.132)	**0.635** (0.132)	2.106 (4.651)	2.107 (4.652)	0.036	0.095
	A4	0.638 (0.133)	0.638 (0.133)	2.152 (4.839)	2.153 (4.839)	0.038	0.097
	B	1.024 (0.023)	1.024 (0.023)	2.681 (5.605)	2.681 (5.605)	0.090	0.178
	C	1.017 (0.027)	1.018 (0.026)	2.257 (4.878)	2.258 (4.880)	-0.038	0.093
	D	0.808 (0.054)	0.808 (0.054)				
2	A1	0.752 (0.066)	0.753 (0.066)	**3.922** (9.992)	**3.924** (10.000)	**0.004**	**0.075**
	A2	0.669 (0.094)	0.669 (0.094)	4.056 (10.551)	4.056 (10.552)	0.032	0.093
	A3	0.648 (0.104)	0.648 (0.104)	4.047 (10.497)	4.048 (10.498)	0.049	0.105
	A4	**0.646** (0.103)	**0.646** (0.102)	4.027 (10.440)	4.028 (10.441)	0.044	0.101
	B	1.012 (0.016)	1.012 (0.016)	4.934 (12.511)	4.935 (12.513)	0.063	0.174
	C	1.010 (0.017)	1.011 (0.016)	4.190 (10.397)	4.192 (10.405)	-0.039	0.104
	D	0.831 (0.035)	0.832 (0.035)				
2.5	A1	0.801 (0.052)	0.802 (0.052)	**2.764** (7.826)	**2.764** (7.828)	**-0.003**	**0.076**
	A2	0.737 (0.069)	0.737 (0.069)	2.790 (8.113)	2.790 (8.113)	0.012	0.082
	A3	**0.715** (0.076)	**0.715** (0.076)	2.815 (8.181)	2.816 (8.181)	0.023	0.089
	A4	0.717 (0.077)	0.717 (0.077)	2.805 (8.142)	2.805 (8.142)	0.019	0.087
	B	1.014 (0.012)	1.014 (0.012)	3.422 (9.565)	3.422 (9.565)	0.079	0.210
	C	1.011 (0.013)	1.011 (0.013)	3.064 (8.548)	3.066 (8.554)	-0.036	0.117
	D	0.869 (0.026)	0.869 (0.026)				

Method A1-A4: penMCFM (EM) for $\alpha_{Enet} = 0.1, 0.5, 0.9, 1$, B: penMCFM (GMIFS), C: MCM (GMIFS), D: penCox.1se; The best result appears in bold.

Table S2: Simulation studies results for $\mathbf{b}_p$, $\boldsymbol{\beta}_p$ and $\pi(\mathbf{z})$ when $\rho = 0.5$

		$\boldsymbol{\beta}_p$		$\mathbf{b}_p$		$\pi(\mathbf{z})$	
v	Method	RME(SD)	ERR (SD)	RME(SD)	ERR (SD)	Bias	MSE
0.5	A1	1.232 (0.244)	1.255 (0.270)	0.396 (0.906)	0.402 (0.923)	**0.007**	0.067
	A2	1.514 (0.950)	1.549 (1.001)	0.421 (0.927)	0.426 (0.934)	0.012	0.070
	A3	1.556 (1.288)	1.593 (1.352)	0.431 (0.940)	0.438 (0.954)	0.013	0.071
	A4	1.454 (0.869)	1.485 (0.918)	0.420 (0.938)	0.426 (0.950)	0.012	0.071
	B	**1.202** (0.049)	**1.203** (0.049)	0.421 (0.959)	0.421 (0.961)	0.232	0.127
	C	1.528 (0.359)	1.530 (0.349)	**0.366** (0.847)	**0.379** (0.875)	-0.022	**0.056**
	D	1.424 (1.859)	1.429 (1.816)				
1	A1	0.812 (0.119)	0.831 (0.111)	1.401 (2.072)	1.423 (2.103)	**0.010**	0.084
	A2	0.798 (0.149)	0.819 (0.145)	1.432 (2.093)	1.451 (2.124)	0.021	0.094
	A3	0.791 (0.151)	0.811 (0.145)	1.460 (2.134)	1.481 (2.165)	0.026	0.099
	A4	**0.788** (0.151)	**0.809** (0.145)	1.457 (2.129)	1.478 (2.162)	0.028	0.100
	B	1.061 (0.030)	1.062 (0.028)	1.795 (2.638)	1.797 (2.640)	0.115	0.154
	C	1.064 (0.048)	1.078 (0.048)	**1.332** (1.879)	**1.366** (1.942)	-0.032	**0.079**
	D	0.829 (0.083)	0.841 (0.081)				
1.5	A1	0.714 (0.080)	0.726 (0.078)	2.658 (6.115)	2.675 (6.123)	**0.011**	**0.078**
	A2	0.634 (0.108)	0.644 (0.108)	**2.652** (5.995)	**2.668** (6.003)	0.035	0.092
	A3	**0.620** (0.119)	**0.629** (0.119)	2.713 (5.951)	2.727 (5.955)	0.043	0.100
	A4	0.625 (0.124)	0.634 (0.123)	2.721 (5.941)	2.735 (5.945)	0.044	0.103
	B	1.022 (0.019)	1.024 (0.018)	3.467 (7.430)	3.469 (7.430)	0.086	0.175
	C	1.010 (0.029)	1.020 (0.024)	2.955 (7.087)	2.972 (7.092)	-0.037	0.091
	D	0.800 (0.054)	0.808 (0.052)				
2	A1	0.758 (0.062)	0.766 (0.062)	3.348 (6.477)	3.367 (6.511)	**0.011**	**0.082**
	A2	0.668 (0.087)	0.674 (0.087)	**3.337** (6.545)	**3.353** (6.570)	0.051	0.107
	A3	0.649 (0.086)	0.654 (0.087)	3.352 (6.573)	3.366 (6.592)	0.062	0.113
	A4	**0.642** (0.089)	**0.647** (0.089)	3.344 (6.556)	3.358 (6.575)	0.063	0.113
	B	1.014 (0.017)	1.017 (0.016)	4.113 (7.934)	4.119 (7.943)	0.056	0.186
	C	1.003 (0.020)	1.011 (0.018)	3.721 (7.153)	3.745 (7.205)	-0.037	0.102
	D	0.828 (0.036)	0.833 (0.035)				
2.5	A1	0.800 (0.051)	0.808 (0.049)	3.588 (8.465)	3.606 (8.510)	**0.002**	**0.078**
	A2	0.734 (0.058)	0.738 (0.059)	3.501 (8.299)	3.516 (8.323)	0.024	0.089
	A3	0.712 (0.072)	0.716 (0.072)	3.457 (8.289)	3.473 (8.306)	0.037	0.098
	A4	**0.705** (0.072)	**0.709** (0.073)	**3.429** (8.211)	**3.445** (8.228)	0.042	0.101
	B	1.014 (0.013)	1.016 (0.011)	4.476 (10.818)	4.479 (10.820)	0.080	0.212
	C	1.005 (0.015)	1.011 (0.012)	3.909 (9.191)	3.931 (9.252)	-0.037	0.109
	D	0.865 (0.028)	0.868 (0.027)				

Method A1-A4: penMCFM (EM) for $\alpha_{Enet} = 0.1, 0.5, 0.9, 1$, B: penMCFM (GMIFS), C: MCM (GMIFS), D: penCox.1se; The best result appears in bold.

2 Additional plots for the Simulation Study

In our study, since we use the EM algorithm to obtain the maximum likelihood estimates of the unknown parameters, the standard errors of the estimated parameters are not directly available, similarly to the approach used in the *smcure* R package and its accompanying paper by Cai et al. [6]. In their work, they used the bootstrap method [13] to estimate the variance of the estimates. While this approach can be effective when dealing with a few covariates, calculating the standard errors in high-dimensional studies is more complex due to the increased number of parameters and potential issues with overfitting.

Our primary objectives in this study are variable selection and the estimation/prediction of the cured/uncured rates, with the ultimate goal of identifying new biomarkers when using the proposed method on genomic/transcriptomic data. In consideration of our objectives, applying the bootstrap method to each dataset in our simulation study does not appear to be worth the effort, as it would be highly time-consuming due to the thousands of variables involved in parameter tuning with cross-validation in the EM algorithm.

Instead, we can provide an empirical estimate of the variability of the estimates based on 100 repeated simulations for each scenario. Specifically, we compute the empirical standard error of the parameter estimates for the 20 non-zero coefficients for both $\boldsymbol{\beta}_p$ and $\mathbf{b}_p$ across the 100 repeatedly simulated datasets. We draw the average estimates of these parameters, and plot them along with one standard error wide bars for some selected cases. These plots are given in Figures (S1) and (S2). Moreover, the same plot is also presented for the uncured rate estimates in Figure (S3), where specifically we plot $\pi(\mathbf{z})$ along with one standard error wide bars, and additionally we plot the 95% CIs is in Figure (S4).

From Figure (S1) we can observe that, for the case $v = 0.5$, our main method penMCFM exhibits a larger standard error compared to the others, and this pattern persists when v is larger, though the differences diminish. The maximum value of the standard errors for penMCFM is 0.0666, while it is 0.0354 for MCM(GMIFS) in this figure. On the other hand, when comparing the estimated values of the coefficients with the true value v on the y-axis, we can observe much less bias in the estimation for penMCFM than for the other methods, as penMCFM always provides values closer to v. Moreover, if considering the variable selection performance of these two methods based on Figure 1 in the manuscript, the MCM(GMIFS) method has many non-zero coefficient estimates with very low sensitivity. Thus, we can conclude that the penMCFM method demonstrates acceptable performance.

Figure (S2) shows a similar pattern, as the overall comparison of the methods is very close to what already observed in Figure (S1). Also here it is evident that penMCFM generally exhibits slightly less biased values of the coefficients as compared to MCM(GMIFS). Since the uncured rate estimate $\hat{\pi}(\mathbf{z})$ is computed based on the coefficient estimates $\mathbf{b}$, having less bias in the coefficients estimates actually helps in the estimation of other parameters in the model. Obviously also the inspection of the behavior of $\hat{\pi}(\mathbf{z})$ can provide a basis for a better comparison. As can be seen in Figure (S3), the standard error values for the penMCFM and MCM(GMIFS) methods are quite similar. However, as shown in Table 1 of the manuscript and Tables S1–S2, the $\hat{\pi}(\mathbf{z})$ estimates from penMCFM generally exhibit lower bias compared to those from MCM(GMIFS), resulting in estimates that are closer to the true values. Given that this parameter represents the uncured/cured rate estimates for the cancer patients in the study, its accuracy is particularly important within the context of our analysis. Thus, we conclude that the penMCFM method demonstrates satisfactory performance under these conditions.

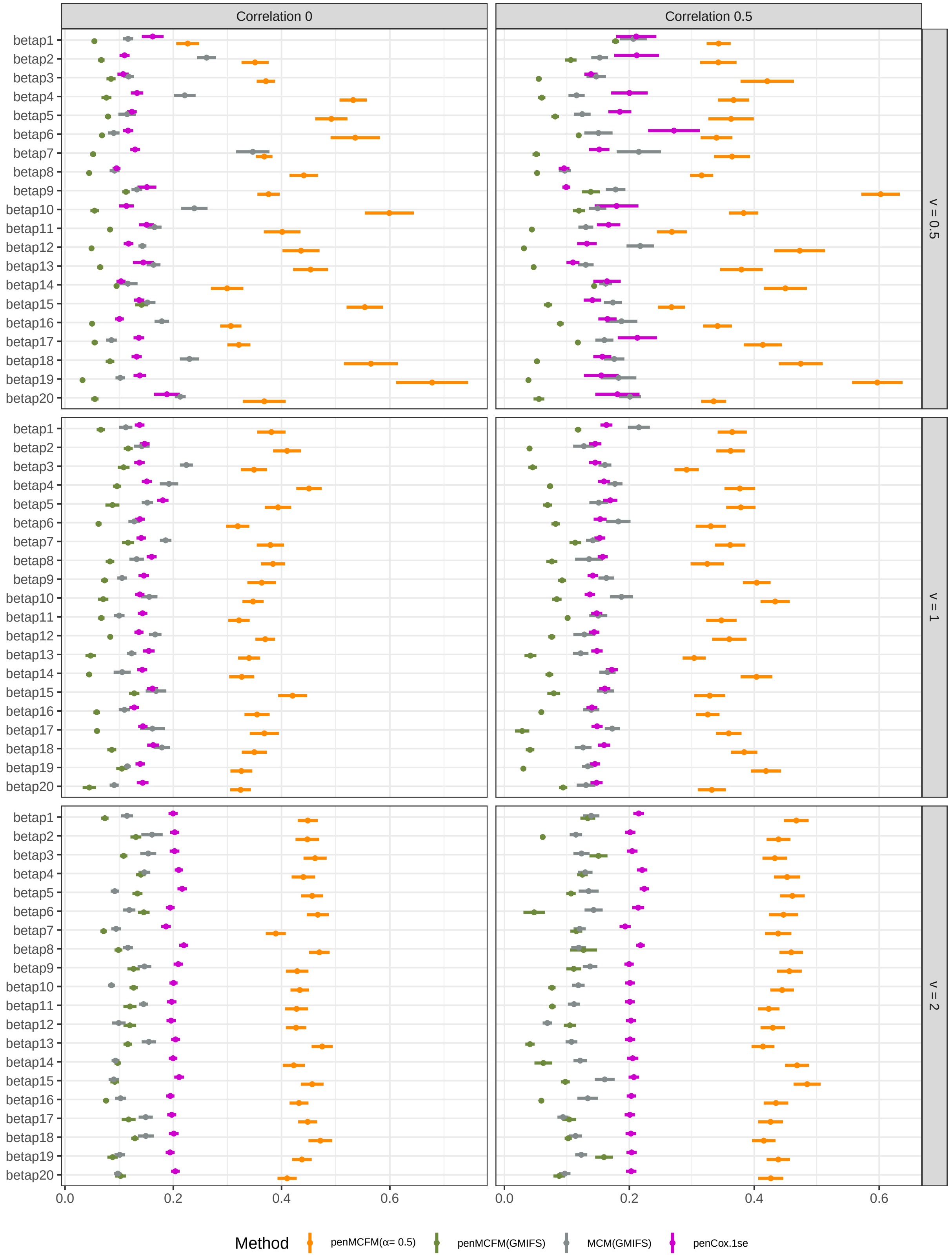

Figure S1: Plots for the average estimates of the non-zero coefficients of $\boldsymbol{\beta}_p$ along with one standard error wide bars

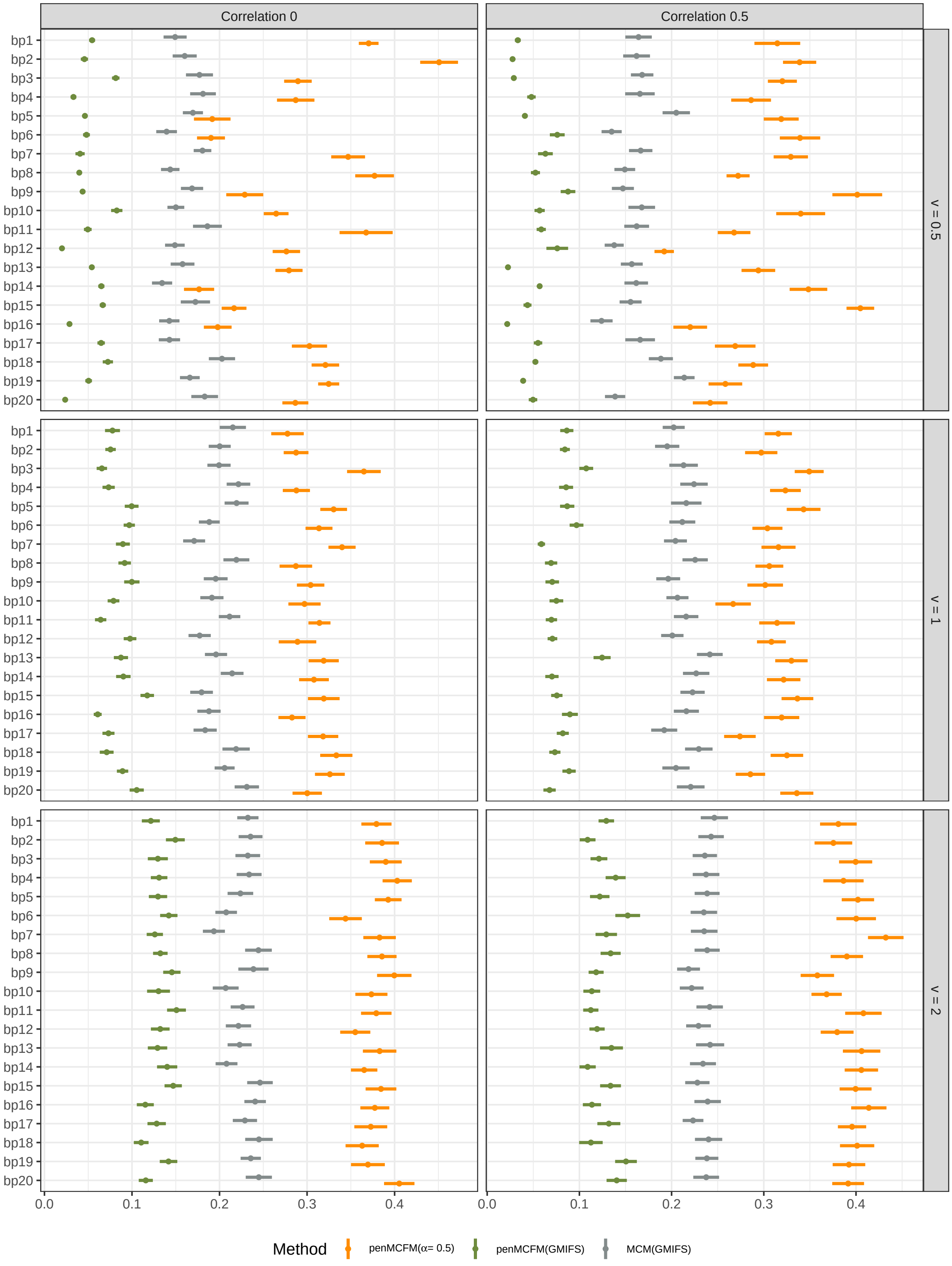

Figure S2: Plots for the average estimates of the non-zero coefficients of $\mathbf{b}_p$ along with one standard error wide bars

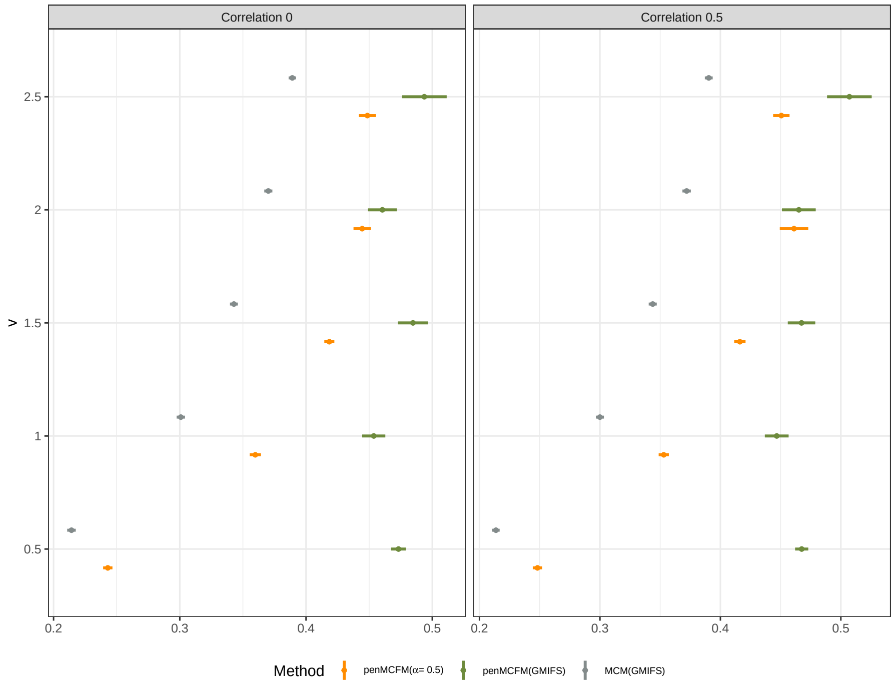

Figure S3: Plots for the average estimates of the uncured rate estimates $\pi(\mathbf{z})$ along with one standard error wide bars, with respect to v and ρ (v represents the true value of the non-zero $\mathbf{b}_p$ coefficients)

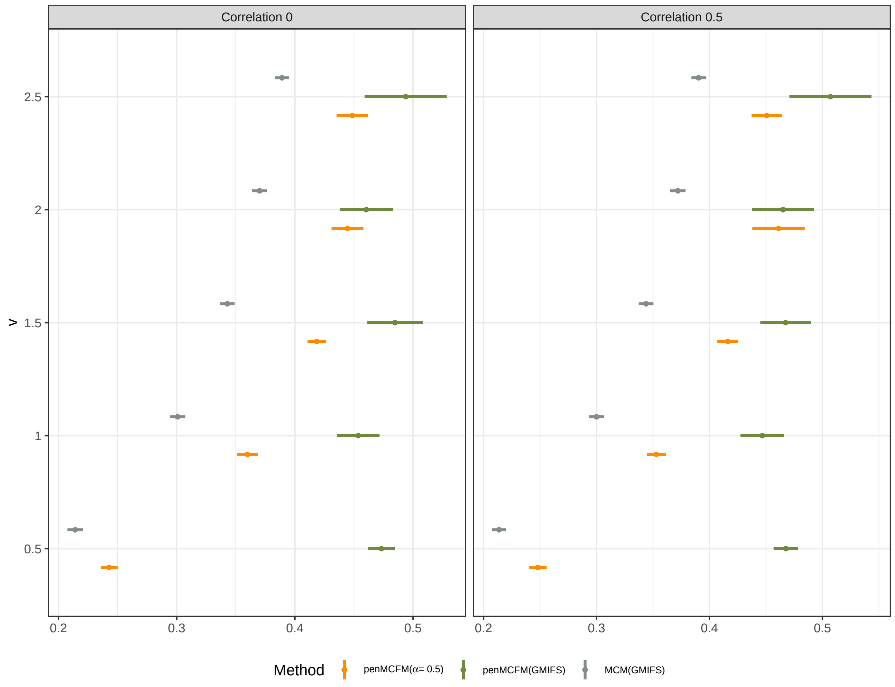

Figure S4: 95% confidence interval of the uncured rate estimates $\pi(\mathbf{z})$

3 Additional figures showing results of the analysis of TCGA-BRCA RNA-Seq data

In Figure S5, we present the overlap of the selected genes for the incidence part of the model across the two GMIFS methods.

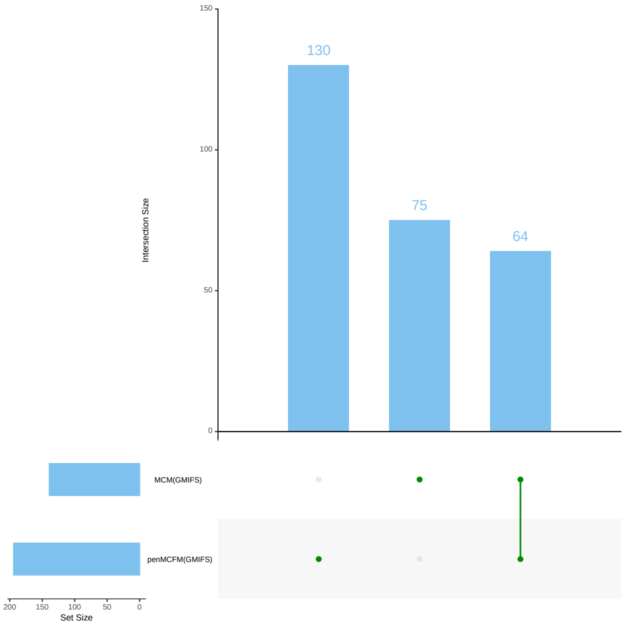

(a) Scenario 1

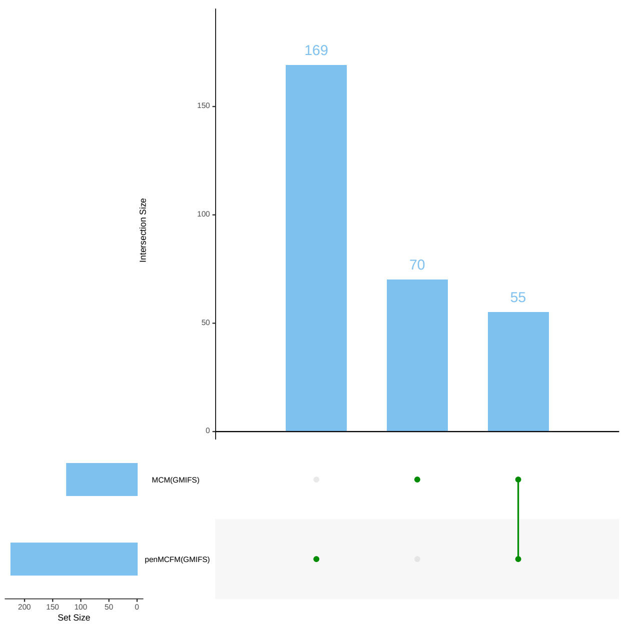

(b) Scenario 2

Figure S5: Results of the analysis of the TCGA-BRCA data. Overlap of the selected gene sets (nonzero $\mathbf{b}_p$ coefficients) between two GMIFS methods: the blue barplots report the frequencies of intersections between the methods, while the bottom green lines report which methods are considered for the overlap

3.1 Results for Scenario 1

3.1.1 penMCFM(GMIFS): enrichment analysis results

In Figure S6, we present GO-EA and KEGG-EA for the incidence part of the model based on penMCFM(GMIFS). For the latency part, we have only one molecular function identified through GO-EA, namely the “histone deacetylase binding (GO:0042826)”. On the other hand, no significant enrichment terms are identified in KEGG-EA.

3.1.2 MCM(GMIFS): enrichment analysis results

For the incidence part, we detect only one significant enriched GO term, namely the “GO:0001533 cornified envelope”, and no significant enrichment terms are identified in KEGG-EA. For the latency part, the significantly enriched GO-EA and KEGG-EA pathway terms are given in Figure S7.

3.1.3 penCox.1se: enrichment analysis results

When focusing on the selected genes based on the penCox.1se method, no significant enrichment terms are identified in either GO-EA or KEGG-EA.

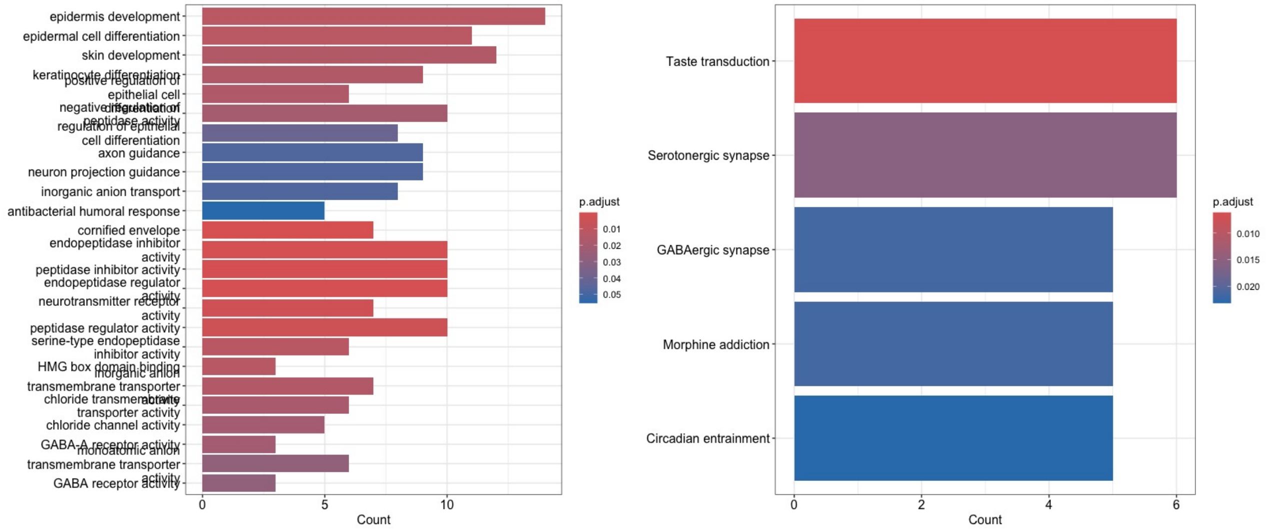

(a) (b)

Figure S6: Results of the analysis of the TCGA-BRCA data. GO-EA and KEGG-EA for the incidence part of the model based on penMCFM(GMIFS): (a) Barplot of significantly enriched GO terms (b) Barplot of significantly enriched KEGG terms

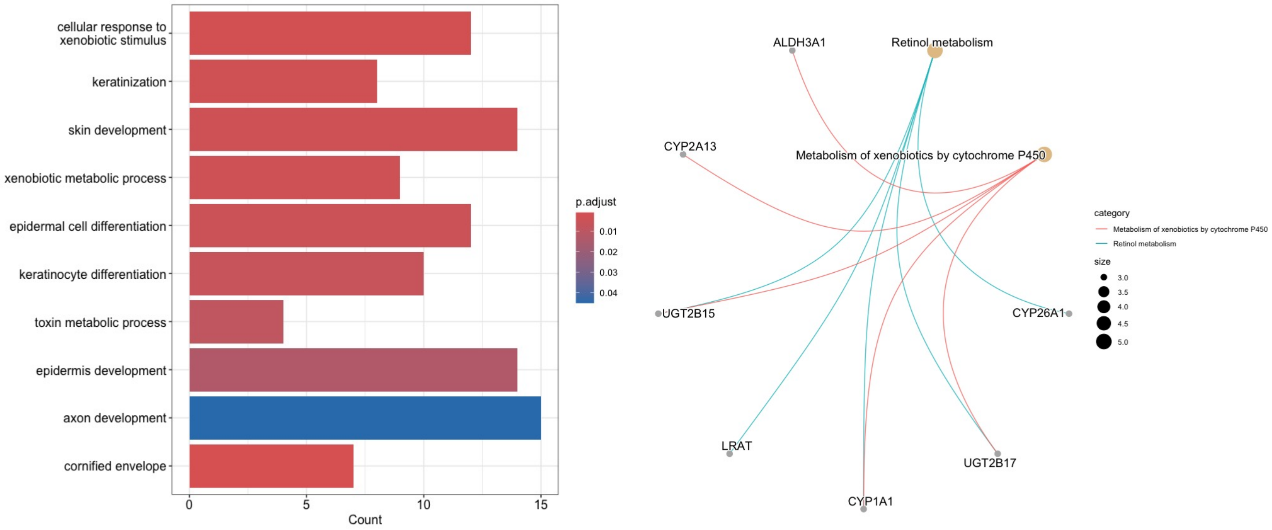

(a) (b)

Figure S7: Results of the analysis of the TCGA-BRCA data. GO-EA and KEGG-EA for the latency part of the model based on MCM(GMIFS): (a) Barplot of significantly enriched GO terms (b) Network plot of enriched KEGG pathway terms and related selected genes

3.2 Results for Scenario 2

3.2.1 penMCFM(EM): additional enrichment analysis results

Additional EA results and obtained breast cancer pathway enriched by selected biomarkers for the penMCFM(EM) are presented in Figures S8-S9.

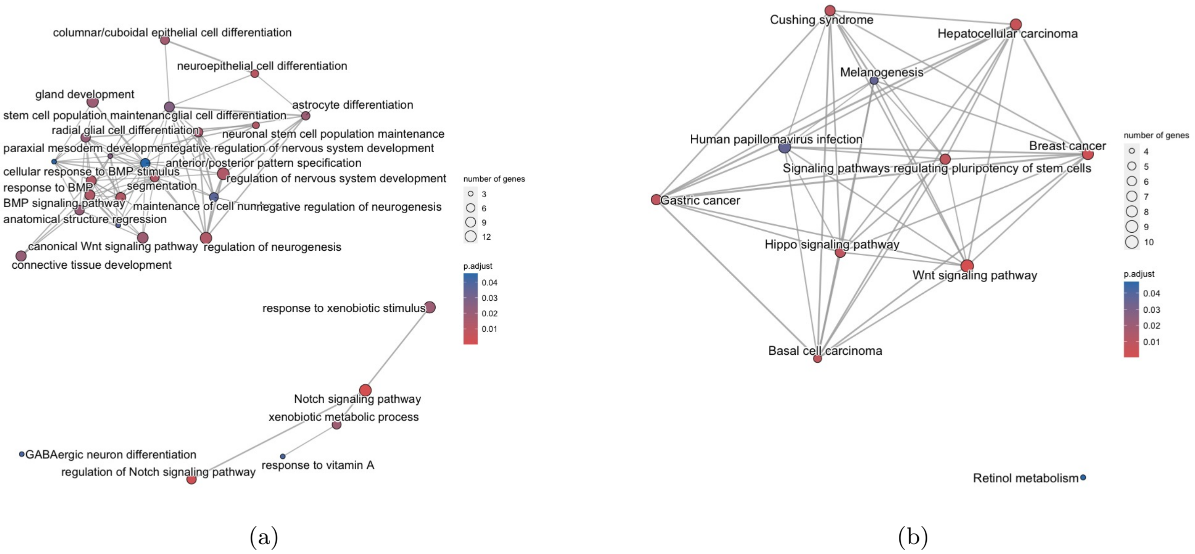

Figure S8: Results of the analysis of the TCGA-BRCA data. (a) Network plot of enriched GO terms (b) Network plot of enriched KEGG pathway terms

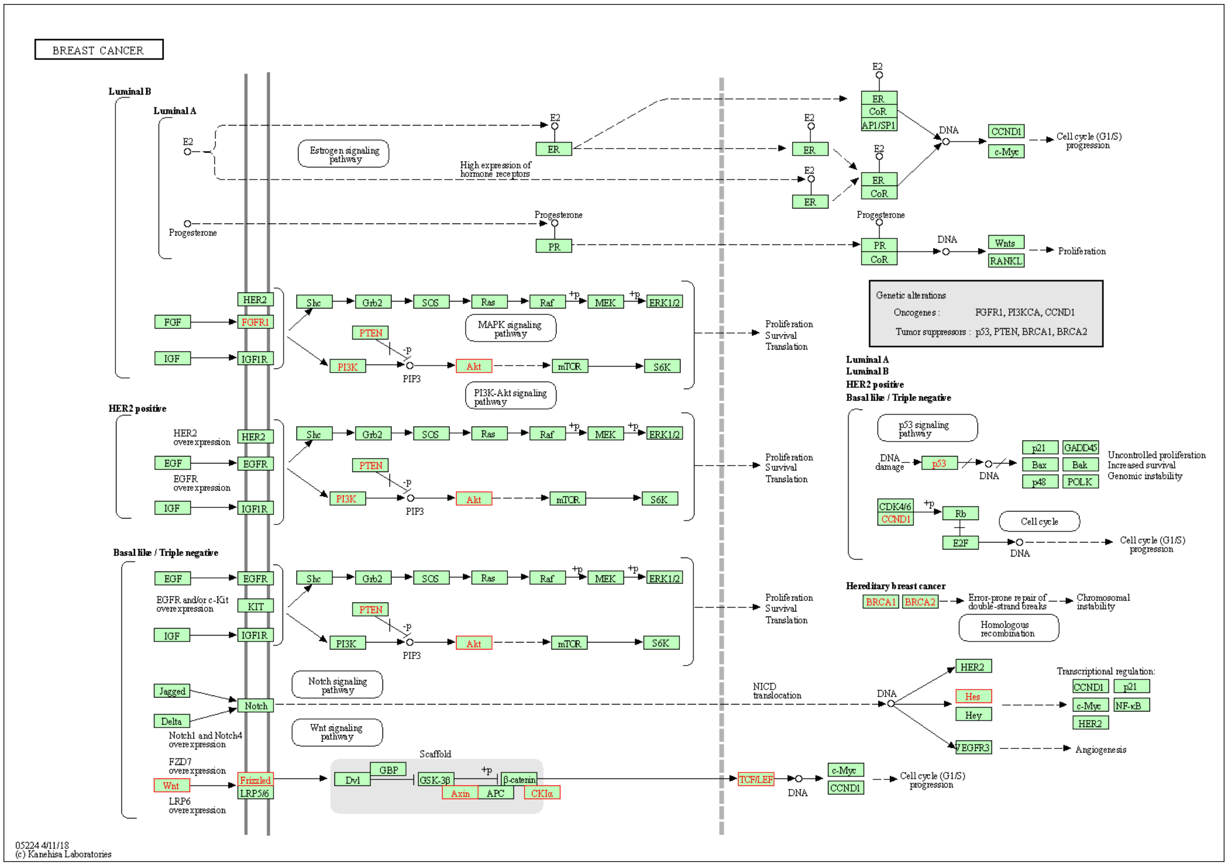

Figure S9: Results of the analysis of the TCGA-BRCA data. The Breast Cancer pathway is one of the top pathways enriched by selected biomarkers obtained when using penMCFM(EM).

3.2.2 penMCFM(GMIFS): enrichment analysis results

For the latency part, the network plot of enriched GO terms and related selected genes is presented in Figure S10. We have not identified any enriched KEGG-EA pathway terms. For the incidence part, GO-EA and KEGG-EA results are given in S11, and also the breast cancer pathway is presented in Figure S12.

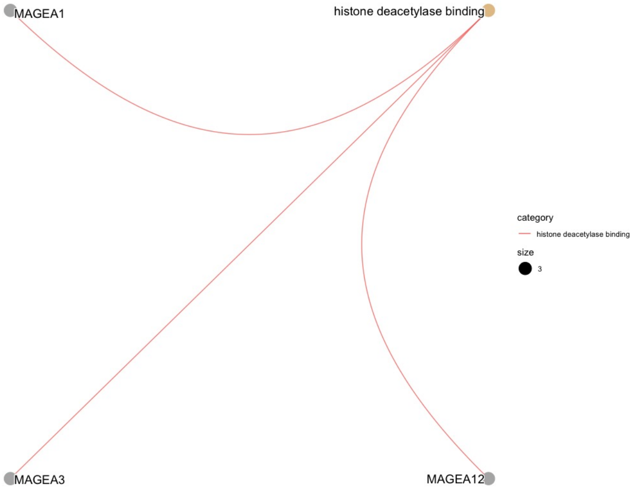

Figure S10: Results of the analysis of the TCGA-BRCA data. Network plot of enriched GO terms and related selected genes for penMCFM(GMIFS)

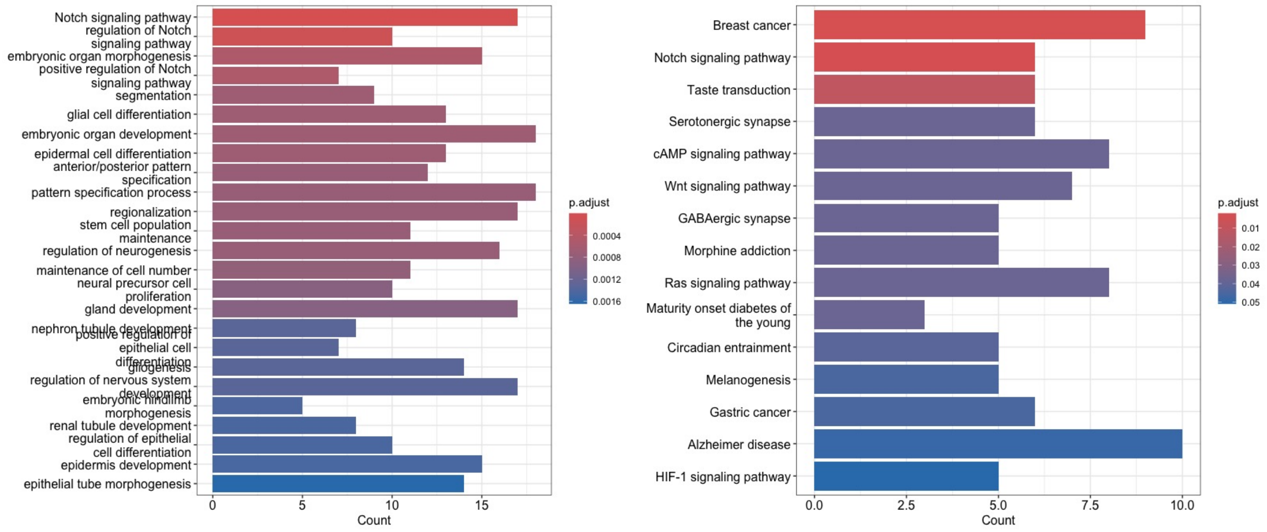

(a) (b)

Figure S11: Results of the analysis of the TCGA-BRCA data. GO-EA and KEGG-EA for the incidence part of the model based on penMCFM(GMIFS): (a) Barplot of significantly enriched GO terms (b) Barplot of significantly enriched KEGG terms

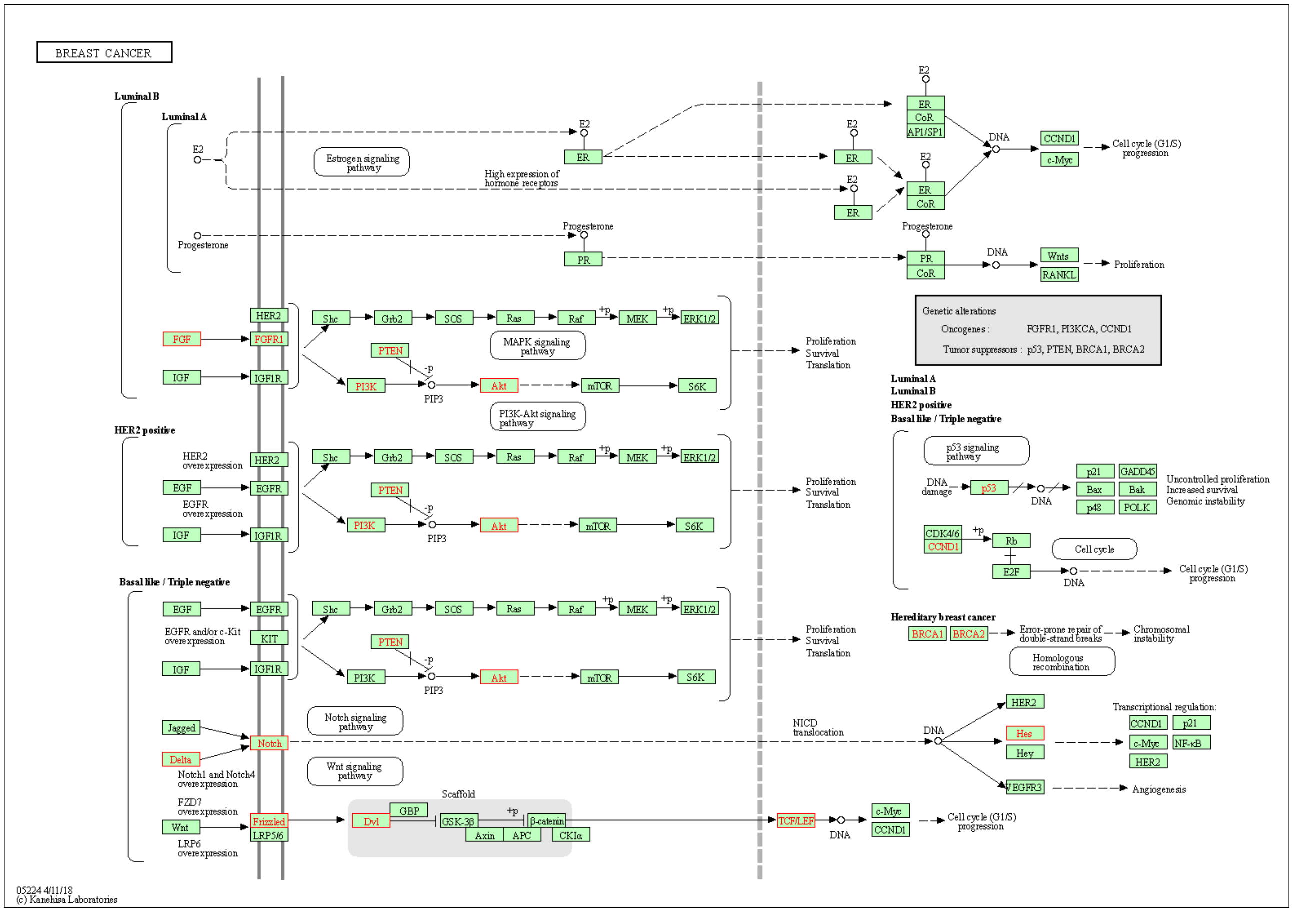

Figure S12: Results of the analysis of the TCGA-BRCA data. The Breast Cancer pathway is the one of top pathways enriched by selected biomarkers obtained from the incidence part of the model when using penM-CFM(GMIFS).

3.2.3 MCM(GMIFS): enrichment analysis results

For the latency part, GO-EA and KEGG-EA results, and the obtained breast cancer pathway are presented in Figures S13-S14. For the incidence part, the barplot of significantly enriched GO terms is given in Figure S15. We have not identified any enriched KEGG-EA pathway terms.

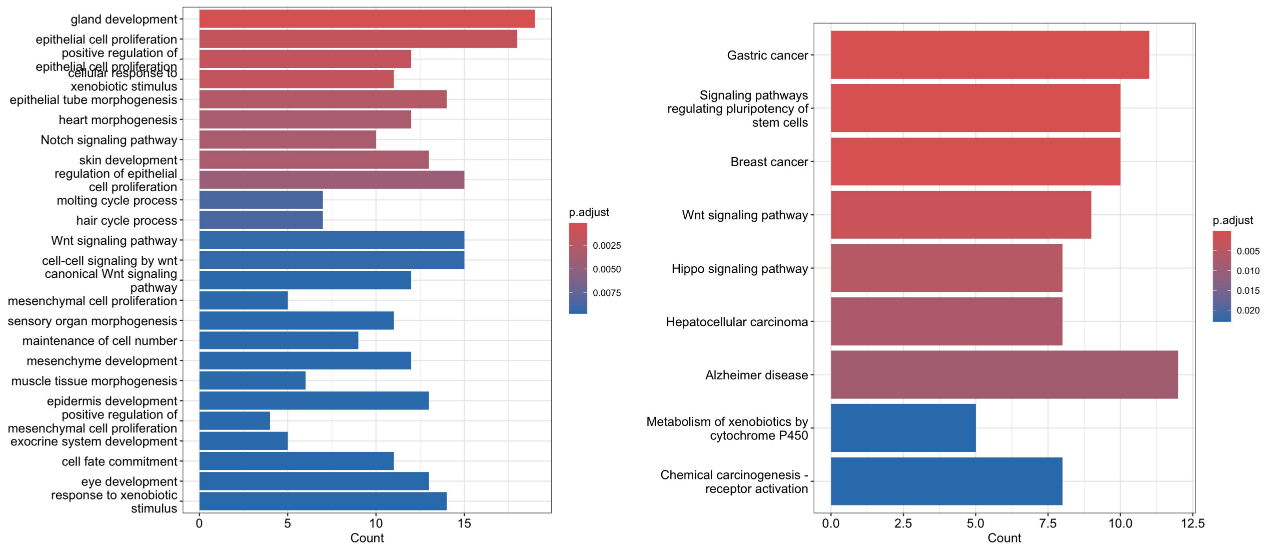

(a) (b)

Figure S13: Results of the analysis of the TCGA-BRCA data. GO-EA and KEGG-EA for the latency part of the model based on MCM(GMIFS): (a) Barplot of significantly enriched GO terms (b) Barplot of significantly enriched KEGG terms

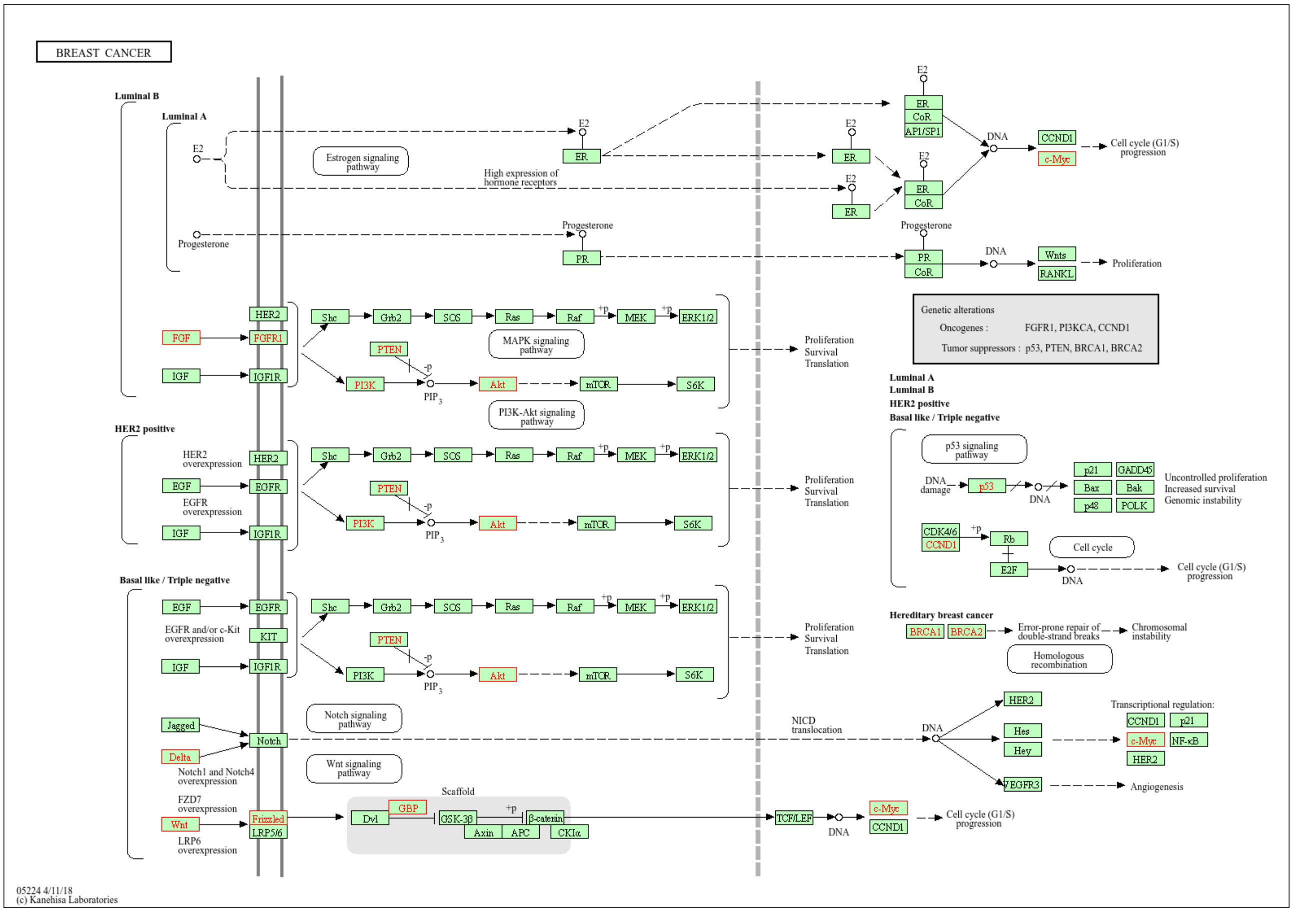

Figure S14: Results of the analysis of the TCGA-BRCA data. The Breast Cancer pathway is the one of top pathways enriched by selected biomarkers obtained from the latency part of the model when using MCM(GMIFS).

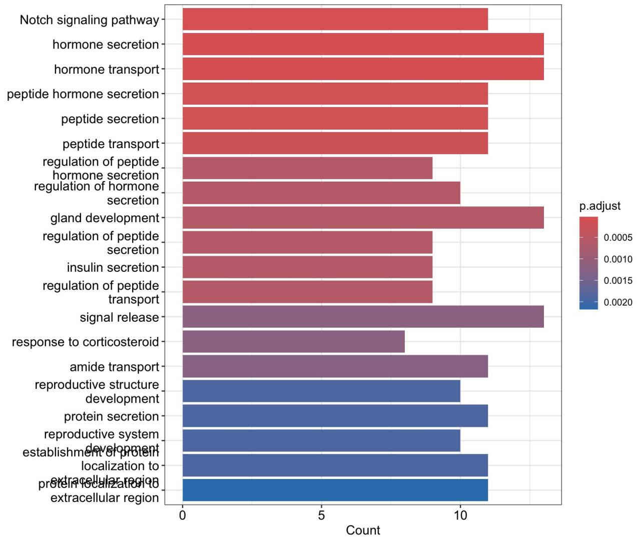

Figure S15: Results of the analysis of the TCGA-BRCA data. GO-EA for the incidence part of the model based on MCM(GMIFS): Barplot of significantly enriched GO terms

3.2.4 penCox.1se: enrichment analysis results

In Figures S16-S17, we present GO-EA and KEGG-EA results, and the related breast cancer pathway.

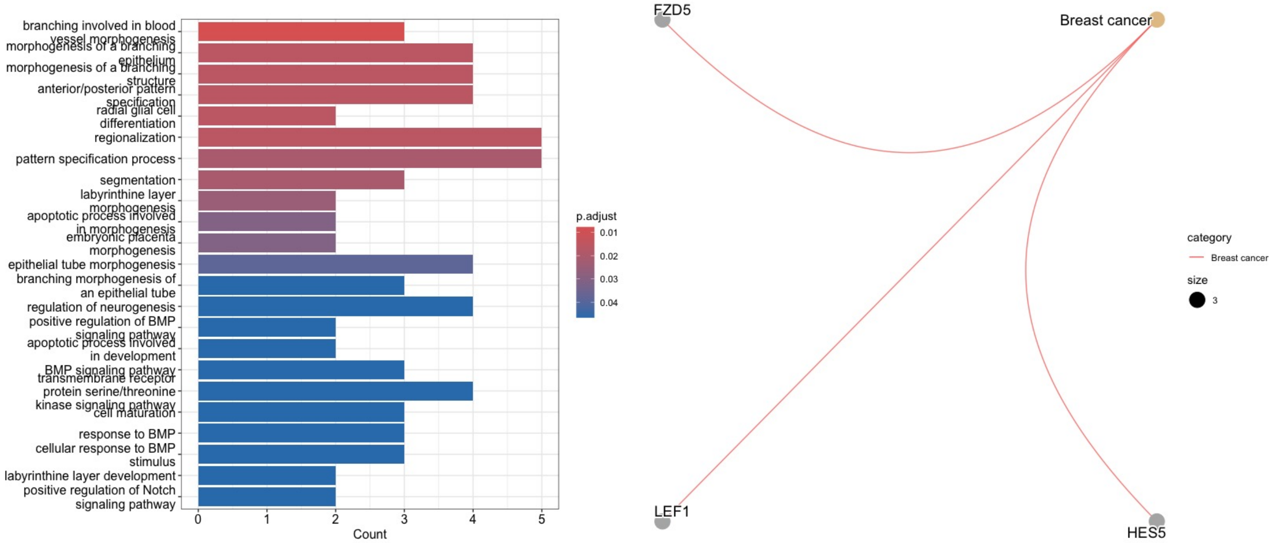

(a) (b)

Figure S16: Results of the analysis of the TCGA-BRCA data. GO-EA and KEGG-EA for the latency part of the model based on penCox.1se: (a) Barplot of significantly enriched GO terms (b) Network plot of enriched KEGG pathway terms and related selected genes

3.2.5 Additional results for the prognostic risk score analyses

The KM curves of low and high risk groups are presented in Figure S18 for penMCFM(GMIFS) and penCox.1se. The heatmaps of the expression values of the selected genes via penMCFM(EM) and MCM(GMIFS) based on the validation dataset are presented in Figure S19.

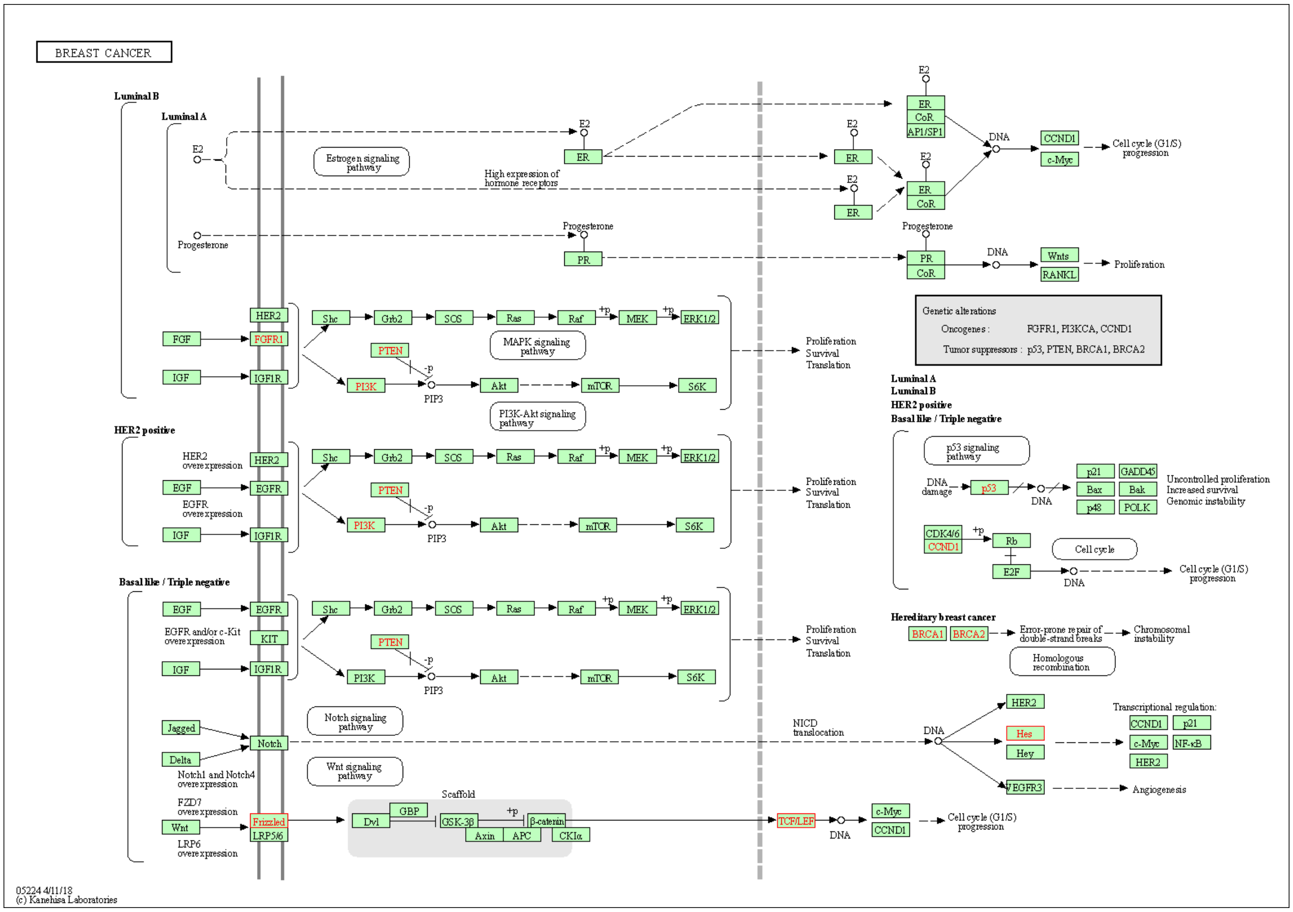

Figure S17: Results of the analysis of the TCGA-BRCA data. The Breast Cancer pathway is the one of top pathways enriched by selected biomarkers obtained when using penCox.1se.

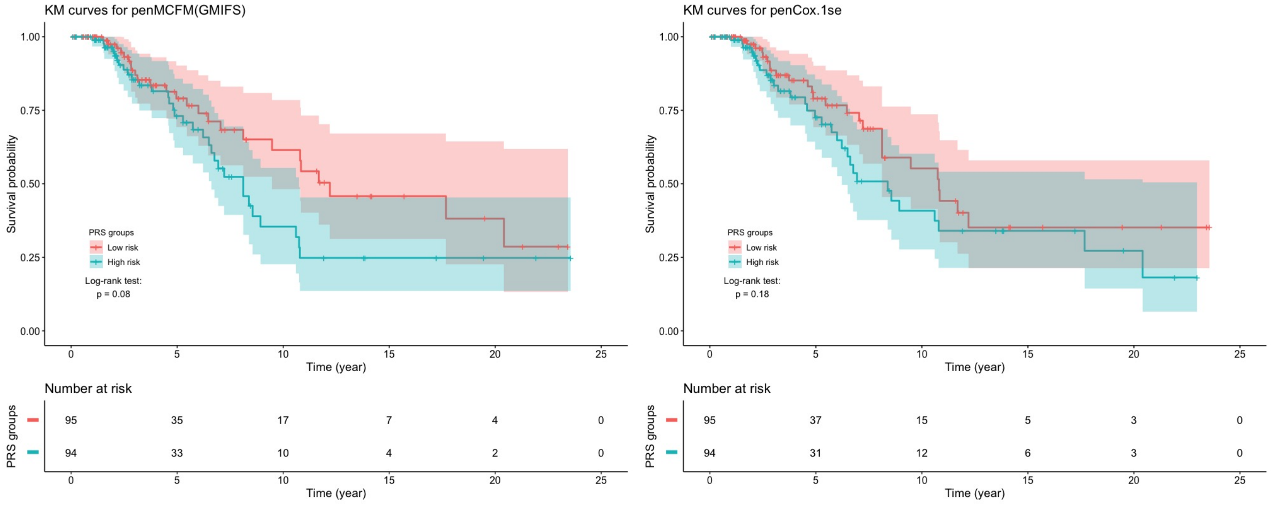

(a) (b)

Figure S18: Results of the analysis of the TCGA-BRCA data. KM curves for the TCGA-BRCA patients in the validation dataset when dichotomized into two groups by the median PRS using the average results of 20 repeats in Scenario 2: (a) penMCFM(GMIFS) and (b) penCox.1se

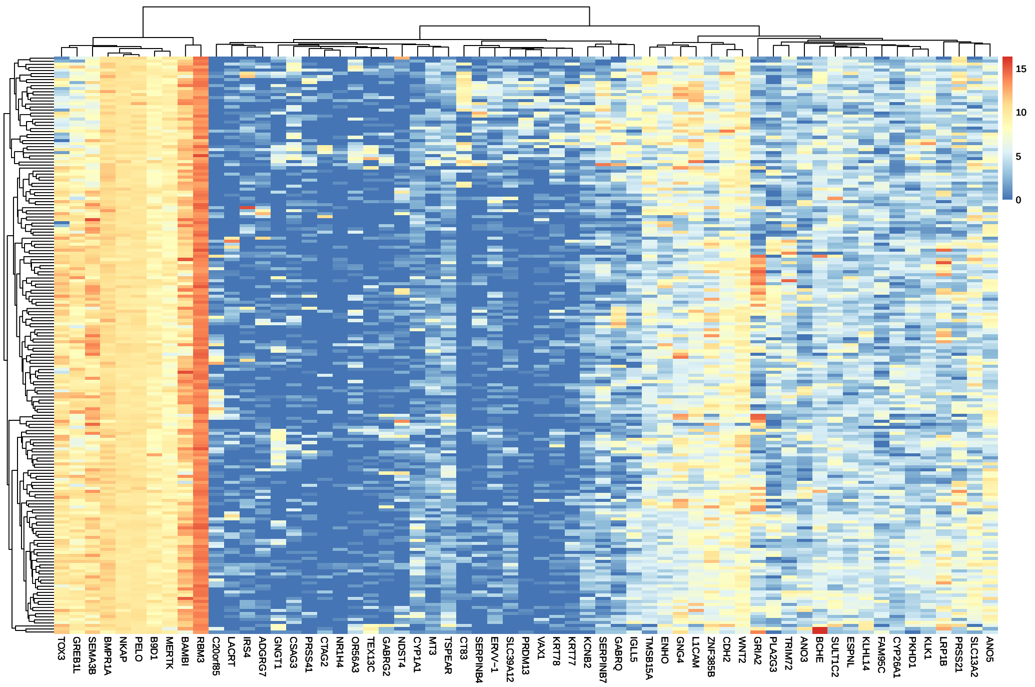

(a)

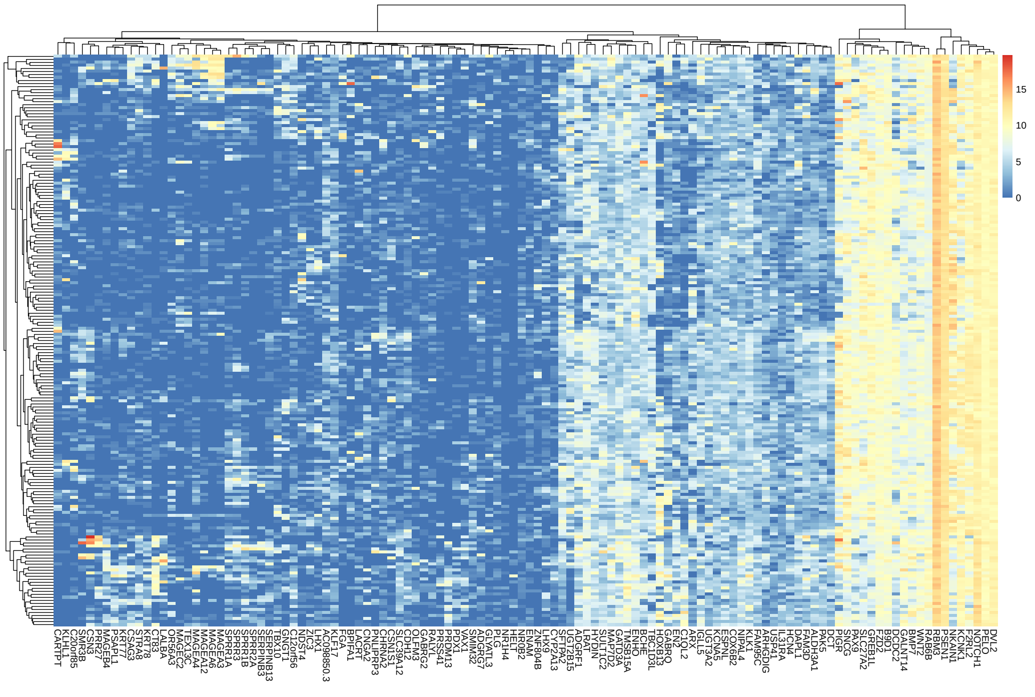

(b)

Figure S19: Results of the analysis of the TCGA-BRCA data. Heatmap of the expression values of the selected genes that are selected in at least 2 over 20 repeats in Scenario 2 for the validation dataset: (a) penMCFM(EM) and (b) MCM(GMIFS)

Table S3: Additional information on the genes list selected by penMCFM(EM) when considering the intersection of results from both scenarios, also in relation to the existing literature on BRCA.

Biomarker	Description	Reported literature	KEGG pathway
IGLL5	The IGLL5 gene can serve not only as a prognostic biomarker for breast cancer patients but also as a potential source of therapeutic strategies for preventing breast cancer recurrence. Its expression is also correlated with tumor-infiltrating immune cells.	[2, 22, 28, 43]	
GABRQ	It has been demonstrated that the mRNA expression of GABRQ could serve as a prognostic marker for clear cell renal cell carcinoma and colon adenocarcinoma. It has been identified as one of the upregulated genes in triple-negative breast cancer (TNBC).	[21, 44, 24]	✓
L1CAM	L1CAM is expressed in numerous human cancers and is frequently associated with poor prognosis. It is suggested that L1CAM can be utilized for breast cancer diagnosis, indicating a potential correlation between L1CAM expression and the overall adverse prognosis of TNBC.	[10, 1, 3]	✓
ADGRG7	The role of this gene in head and neck squamous cell carcinoma, uterine corpus endometrial carcinoma, and breast cancers are investigated. It is observed that the gene is directly involved in breast tumor metastasis to bone tissues.	[31, 23, 38]	
VAX1	It has been identified as one of the 10 upregulated genes in TNBC. Its association with lung squamous cell carcinoma has also been observed, suggesting its potential as a prognostic biomarker for evaluating risk assessment.	[16, 26]	
CSAG3	CSAG3, also known as TRAG-3 (Taxol Resistance Associated Gene-3), is reported to be upregulated in numerous tumors, including gastric cancer, urothelial carcinoma of the bladder, ovarian carcinoma, and melanoma.	[46]	
ENHO	Adropin is encoded by the energy homeostasis-associated (ENHO) gene. Its association with the development of colorectal cancer has also been investigated. GPR19 is activated by adropin is studied for the breast tumor cells.	[36, 20]	
OR56A3	The Olfactory Receptor (OR) family has gained attention as a potential biomarker for cancer. The significance of transcript abundance in certain OR genes is examined in an invasive breast carcinoma population. However, a substantial portion of the roles of OR genes in breast cancer remains understudied.	[41, 30]	✓
C20orf85	It has been implicated in various cancer types, including lung cancer, lower-grade glioma of the brain, ovarian cancer, and breast cancer. Further investigations are needed to explore its implications.	[18, 37, 4, 15]	
GREB1L	It has been observed that the GREB1L gene is implicated in the development of breast cancer, and it is proposed as a potential molecular marker for predicting the prognosis of breast cancer. It has been also demonstrated to exhibit a high correlation with both estrogen receptor and androgen receptor expression in breast/prostate cancer cell lines and primary tumors.	[5, 11]	
BMPR1A	The Bone Morphogenetic Protein Receptor (BMPR) genes associated with various cancer types is explored. The role of the BMPR1A gene in breast cancer growth and metastasis is also investigated.	[34, 35, 17]	✓
TSPEAR	The TSPEAR gene has not been reported in any cancer study, except for one preprint study related to colorectal cancer. Further investigations are required to delve into its implications.	[27]	
KRT77	The differential expression of KRT genes is investigated across various cancers. Its potential role as a biomarker in head and neck squamous cell carcinoma is also explored.	[9, 39]	

Table S3 Continued.

Biomarker	Description	Reported literature	KEGG pathway
GNG4	It has been reported as a potential biomarker in various cancer types, including bladder, colorectal, gastric, and breast cancer.	[47, 29, 12, 3]	✓
SERPINB7	The SERPINB family genes are differentially expressed in the tumor tissues. The SERPINB7 gene has been studied in various cancer types, including breast, cervical, and lung cancers.	[8, 42, 33]	
GABRG2	GABA receptor genes (GABR) constitute a group of genes associated with developmental and epileptic encephalopathies. They have also been identified to be associated with recurrent breast, colon, and laryngeal cancer samples.	[19, 40, 44, 32]	✓
ZNF385B	The potential effects of ZNF385B expression in breast cancer have been determined in recent studies, suggesting its utility as a potential diagnostic and prognostic biomarker for breast cancer. It is also observed to be correlated with the overall survival of ovarian cancer patients.	[14, 45, 49]	
CT83	CT83 is highly expressed in gastric, triple-negative breast, lung, and hepatocellular cancers. It is observed to be significantly associated with the overall survival of TNBC patients.	[48, 7, 25]	

References

[1] Peter Altevogt, Kai Doberstein, and Mina Fogel. L1cam in human cancer. *International Journal of Cancer*, 138(7):1565–1576, 2016.

[2] Maria Libera Ascierto, Maciej Kmieciak, Michael O Idowu, Rose Manjili, Yingdong Zhao, Margaret Grimes, Catherine Dumur, Ena Wang, Viswanathan Ramakrishnan, Xiang-Yang Wang, et al. A signature of immune function genes associated with recurrence-free survival in breast cancer patients. *Breast Cancer Research and Treatment*, 131:871–880, 2012.

[3] Carlos A Barrón-Gallardo, Mariel Garcia-Chagollán, Andres J Morán-Mendoza, Raul Delgadillo-Cristerna, María G Martínez-Silva, María M Villaseñor-García, Adriana Aguilar-Lemarroy, and Luis F Jave-Suárez. A gene expression signature in her2+ breast cancer patients related to neoadjuvant chemotherapy resistance, overall survival, and disease-free survival. *Frontiers in Genetics*, 13:991706, 2022.

[4] Banabithi Bose, Matthew Moravec, and Serdar Bozdag. Computing microrna-gene interaction networks in pan-cancer using mirdriver. *Scientific Reports*, 12(1):3717, 2022.

[5] Patrick D Brophy, Maria Rasmussen, Mrutyunjaya Parida, Greg Bonde, Benjamin W Darbro, Xiaojing Hong, Jason C Clarke, Kevin A Peterson, James Denegre, Michael Schneider, et al. A gene implicated in activation of retinoic acid receptor targets is a novel renal agenesis gene in humans. *Genetics*, 207(1):215–228, 2017.

[6] Chao Cai, Yubo Zou, Yingwei Peng, and Jiajia Zhang. smcure: An r-package for estimating semiparametric mixture cure models. *Comput Methods Programs Biomed*, 108(3):1255–1260, 2012.

[7] Chen Chen, Dan Gao, Jinlong Huo, Rui Qu, Youming Guo, Xiaochi Hu, and Libo Luo. Multiomics analysis reveals ct83 is the most specific gene for triple negative breast cancer and its hypomethylation is oncogenic in breast cancer. *Scientific Reports*, 11(1):12172, 2021.

[8] Ruey-Hwang Chou, Hui-Chin Wen, Wei-Guang Liang, Sheng-Chieh Lin, Hsiao-Wei Yuan, Cheng-Wen Wu, and Wun-Shaing Wayne Chang. Suppression of the invasion and migration of cancer cells by serpinb family genes and their derived peptides. *Oncology Reports*, 27(1):238–245, 2012.

[9] A Dhakal, N Mladkova, and DM Blakaj. Divergent role of intermediate filaments in clinical outcomes of hpv-positive and hpv-negative head and neck squamous cell carcinoma. *International Journal of Radiation Oncology, Biology, Physics*, 111(3):e368, 2021.

[10] Kai Doberstein, Karin Milde-Langosch, Niko P Bretz, Uwe Schirmer, Ayelet Harari, Isabell Witzel, Alon Ben-Arie, Michael Hubalek, Elisabeth Müller-Holzner, Susanne Reinold, et al. L1cam is expressed in

triple-negative breast cancers and is inversely correlated with androgen receptor. *BMC cancer*, 14(1):1–13, 2014.

[11] Ke Dong, Chenchen Geng, Xiaohong Zhan, Zhi Sun, Qian Pu, Peng Li, Haiyun Song, Guanghui Zhao, and Haidong Gao. Greb1l overexpression is associated with good clinical outcomes in breast cancer. *European Journal of Medical Research*, 28(1):510, 2023.

[12] Lianhui Duan, Xuefei Liu, Ziwei Luo, Chen Zhang, Chun Wu, Weiping Mu, Zhixiang Zuo, Xiaoqing Pei, and Tian Shao. G-protein subunit gamma 4 as a potential biomarker for predicting the response of chemotherapy and immunotherapy in bladder cancer. *Genes*, 13(4):693, 2022.

[13] Bradley Efron and Robert J Tibshirani. *An introduction to the bootstrap.* Chapman and Hall/CRC, 1994.

[14] Bente Vilming Elgaaen, Ole Kristoffer Olstad, Leiv Sandvik, Elin Ødegaard, Torill Sauer, Anne Cathrine Staff, and Kaare M Gautvik. Znf385b and vegfa are strongly differentially expressed in serous ovarian carcinomas and correlate with survival. *PloS One*, 7:e46317, 2012.

[15] Daniela Furrer, Dzevka Dragic, Sue-Ling Chang, Frédéric Fournier, Arnaud Droit, Simon Jacob, and Caroline Diorio. Association between genome-wide epigenetic and genetic alterations in breast cancer tissue and response to her2-targeted therapies in her2-positive breast cancer patients: new findings and a systematic review. *Cancer Drug Resistance*, 5(4):995, 2022.

[16] Chundi Gao, Jing Zhuang, Chao Zhou, Ke Ma, Minzhang Zhao, Cun Liu, Lijuan Liu, Huayao Li, Fubin Feng, and Changgang Sun. Prognostic value of aberrantly expressed methylation gene profiles in lung squamous cell carcinoma: A study based on the cancer genome atlas. *Journal of Cellular Physiology*, 234(5):6519–6528, 2019.

[17] Adam Hermawan and Herwandhani Putri. Bioinformatics analysis of the genetic and epigenetic alterations of bone morphogenetic protein receptors in metastatic breast cancer. *Biochemical Genetics*, pages 1–27, 2023.

[18] Kyeong-Man Hong, Sei-Hoon Yang, Sinchita Roy Chowdhuri, Audrey Player, Megan Hames, Junya Fukuoka, Daoud Meerzaman, Tatiana Dracheva, Zhifu Sun, Ping Yang, et al. Inactivation of llc1 gene in nonsmall cell lung cancer. *International Journal of Cancer*, 120(11):2353–2358, 2007.

[19] Jianhua Hu. Cancer outlier detection based on likelihood ratio test. *Bioinformatics*, 24(19):2193–2199, 2008.

[20] Linghui Jia, Liting Liao, Yongshuai Jiang, Xiangyu Hu, Guotao Lu, Weiming Xiao, Weijuan Gong, Xiaoqin Jia, and Jia Xiaoqin. Low-dose adropin stimulates inflammasome activation of macrophage via mitochondrial ros involved in colorectal cancer progression. *BMC Cancer*, 23(1):1042, 2023.

[21] Dongjun Lee, Mihyang Ha, Chae Mi Hong, Jayoung Kim, Su Min Park, Dongsu Park, Dong Hyun Sohn, Ho Jin Shin, Hak-Sun Yu, Chi Dae Kim, et al. Gabrq expression is a potential prognostic marker for patients with clear cell renal cell carcinoma. *Oncology Letters*, 18(6):5731–5738, 2019.

[22] Hannah Lee, Mi Jeong Kwon, Beom-Mo Koo, Hee Geon Park, Jinil Han, and Young Kee Shin. A novel immune prognostic index for stratification of high-risk patients with early breast cancer. *Scientific Reports*, 11(1):128, 2021.

[23] Ping Lei, Hongmei Wang, Liting Yu, Cong Xu, Haojie Sun, Yihan Lyu, Lianqin Li, and Dao-Lai Zhang. A correlation study of adhesion g protein-coupled receptors as potential therapeutic targets in uterine corpus endometrial cancer. *International Immunopharmacology*, 108:108743, 2022.

[24] Na Li, Xiang Xu, Dan Liu, Jiaxin Gao, Ying Gao, Xufeng Wu, Huiming Sheng, Qun Li, and Jun Mi. The delta subunit of the gabaa receptor is necessary for the gpt2-promoted breast cancer metastasis. *Theranostics*, 13(4):1355, 2023.

[25] Qingyang Li, Wei Hu, Baoyi Liao, Chanchan Song, and Liangping Li. Natural high-avidity t-cell receptor efficiently mediates regression of cancer/testis antigen 83 positive common solid cancers. *Journal for Immunotherapy of Cancer*, 10(7):e004713, 2022.

[26] Xiaohong Li, Eric C Rouchka, Guy N Brock, Jun Yan, Timothy E O'Toole, David A Tieri, and Nigel GF Cooper. A combined approach with gene-wise normalization improves the analysis of rna-seq data in human breast cancer subtypes. *PloS One*, 13(8):e0201813, 2018.

[27] Xintong Li, Yuandong Xie, Shuoyao Su, Zhe Liu, Jia Zhao, and Dezhong Wen. Increased expression of tspear in colorectal cancer predicts poor prognosis. 2022.

[28] Feng Liang, Hongzhu Qu, Qiang Lin, Yadong Yang, Xiuyan Ruan, Bo Zhang, Yi Liu, Chengze Yu, Hongyan Zhang, Xiangdong Fang, et al. Molecular biomarkers screened by next-generation rna sequencing for non-sentinel lymph node status prediction in breast cancer patients with metastatic sentinel lymph nodes. *World Journal of Surgical Oncology*, 13:1–10, 2015.

[29] Xiao-hong Mao, Qiang Ye, Guo-bing Zhang, Jin-ying Jiang, Hong-ying Zhao, Yan-fei Shao, Zi-qi Ye, Zi-xue Xuan, and Ping Huang. Identification of differentially methylated genes as diagnostic and prognostic biomarkers of breast cancer. *World journal of surgical oncology*, 19:1–11, 2021.

[30] Shirin Masjedi, Laurence J Zwiebel, and Todd D Giorgio. Olfactory receptor gene abundance in invasive breast carcinoma. *Scientific Reports*, 9(1):13736, 2019.

[31] Di Meng, Tongjun Liu, Feng Ma, and Mingguo Wang. Screening the key genes of prognostic value in the microenvironment for head and neck squamous cell carcinoma. *Medicine*, 100(4):1–8, 2021.

[32] Gerald Nwosu, Shilpa B Reddy, Heather Rose Mead Riordan, and Jing-Qiong Kang. Variable expression of gabaa receptor subunit gamma 2 mutation in a nuclear family displaying developmental and encephalopathic phenotype. *International Journal of Molecular Sciences*, 23(17):9683, 2022.

[33] Junwen Ou, Qiulin Liao, Yanping Du, Wentao Xi, Qiong Meng, Kexin Li, Qichun Cai, and Clifford LK Pang. Serpine1 and serpinb7 as potential biomarkers for intravenous vitamin c treatment in non-small-cell lung cancer. *Free Radical Biology and Medicine*, 209:96–107, 2023.

[34] Hannah L O'Neill, Amy P Cassidy, Olivia B Harris, and John W Cassidy. Bmp2/bmpr1a is linked to tumour progression in dedifferentiated liposarcomas. *PeerJ*, 4:e1957, 2016.

[35] Michael W Pickup, Laura D Hover, Yan Guo, Agnieszka E Gorska, Anna Chytil, Sergey V Novitskiy, Harold L Moses, and Philip Owens. Deletion of the bmp receptor bmpr1a impairs mammary tumor formation and metastasis. *Oncotarget*, 6(26):22890, 2015.

[36] Angad Rao and Deron R Herr. G protein-coupled receptor gpr19 regulates e-cadherin expression and invasion of breast cancer cells. *Biochimica et Biophysica Acta (BBA)-Molecular Cell Research*, 1864(7):1318–1327, 2017.

[37] Andrew J Shih, Andrew Menzin, Jill Whyte, John Lovecchio, Anthony Liew, Houman Khalili, Tawfiqul Bhuiya, Peter K Gregersen, and Annette T Lee. Identification of grade and origin specific cell populations in serous epithelial ovarian cancer by single cell rna-seq. *PLoS One*, 13(11):e0206785, 2018.

[38] Yashbir Singh, Naidu Subbarao, Abhinav Jaimini, Quincy A Hathaway, Amina Kunovac, Bradley Erickson, Vishnu Swarup, and Himanshu Narayan Singh. Genome-wide expression reveals potential biomarkers in breast cancer bone metastasis. *Journal of Integrative Bioinformatics*, 19(3):20210041, 2022.

[39] Işıl Takan, Gökhan Karakülah, Aikaterini Louka, and Athanasia Pavlopoulou. "in the light of evolution:" keratins as exceptional tumor biomarkers. *PeerJ*, 11:e15099, 2023.

[40] Zhengyi Tang, Ganguan Wei, Longcheng Zhang, and Zhiwen Xu. Signature micrornas and long noncoding rnas in laryngeal cancer recurrence identified using a competing endogenous rna network. *Molecular Medicine Reports*, 19(6):4806–4818, 2019.

[41] Lea Weber, Désirée Maßberg, Christian Becker, Janine Altmüller, Burkhard Ubrig, Gabriele Bonatz, Gerhard Wölk, Stathis Philippou, Andrea Tannapfel, Hanns Hatt, et al. Olfactory receptors as biomarkers in human breast carcinoma tissues. *Frontiers in Oncology*, 8:33, 2018.

[42] Hua-Fang Wei, Rui-Feng Zhang, Yue-Chen Zhao, and Xian-Shuang Tong. Serpinb7 as a prognostic biomarker in cervical cancer: Association with immune infiltration and facilitation of the malignant phenotype. *Heliyon*, 9(9), 2023.

[43] Zhi-Nan Xia, Xing-Yuan Wang, Li-Cheng Cai, Wen-Gang Jian, and Cheng Zhang. Igll5 is correlated with tumor-infiltrating immune cells in clear cell renal cell carcinoma. *FEBS Open Bio*, 11(3):898–910, 2021.

[44] Ling Yan, Yi-Zhen Gong, Meng-Nan Shao, Guo-Tian Ruan, Hai-Lun Xie, Xi-Wen Liao, Xiang-Kun Wang, Quan-Fa Han, Xin Zhou, Li-Cheng Zhu, et al. Distinct diagnostic and prognostic values of γ-aminobutyric acid type a receptor family genes in patients with colon adenocarcinoma. *Oncology Letters*, 20(1):275–291, 2020.

[45] Ning Yan, Cong Liu, Fang Tian, Ling Wang, Yimin Wang, Zhaoying Yang, Yan Jiao, Miao He, et al. Downregulated mrna expression of znf385b is an independent predictor of breast cancer. *International Journal of Genomics*, 2021, 2021.

[46] Xiuzhi Zhang, Chunyan Kang, Ningning Li, Xiaoli Liu, Jinzhong Zhang, Fenglan Gao, and Liping Dai. Identification of special key genes for alcohol-related hepatocellular carcinoma through bioinformatic analysis. *PeerJ*, 7:e6375, 2019.

[47] Hongcan Zhao, Danli Sheng, Ze Qian, Sunyi Ye, Jianzhong Chen, and Zhe Tang. Identifying gng4 might play an important role in colorectal cancer tmb. *Cancer Biomarkers*, 32(4):435–450, 2021.

[48] Guansheng Zhong, Weiyang Lou, Qinyan Shen, Kun Yu, and Yajuan Zheng. Identification of key genes as potential biomarkers for triple-negative breast cancer using integrating genomics analysis. *Molecular Medicine Reports*, 21(2):557–566, 2020.

[49] Zhenhua Zhong, Wenqiang Jiang, Jing Zhang, Zhanwen Li, and Fengfeng Fan. Identification and validation of a novel 16-gene prognostic signature for patients with breast cancer. *Scientific Reports*, 12(1):12349, 2022.
